# Association between Metrnl and carotid atherosclerosis in patients with type 2 diabetes mellitus

**DOI:** 10.3389/fendo.2024.1414508

**Published:** 2025-01-08

**Authors:** Chunmei Li, Qiongzhu Huang, Yanqian Zhuang, Pin Chen, Yiyang Lin

**Affiliations:** ^1^ Department of Endocrine, The 900th Hospital of Joint Logistic Support Force, the Chinese People's Liberation Army (PLA), Fuzhou, China; ^2^ Department of Endocrine, Fuzong Clinical Medical College of Fujian Medical University, Fuzhou, China

**Keywords:** Metrnl, carotid atherosclerosis, carotid intima-media thickness, type 2 diabetes mellitus, cross-sectional study

## Abstract

**Objective:**

Type 2 diabetes mellitus (T2DM) is a major cause of atherosclerosis, as well as an independent risk factor of cardiovascular adverse events. We aimed to evaluate the association of serum Meteorin-like protein (Metrnl) level with carotid atherosclerosis as determined by carotid intima-media thickness (CIMT) status in subjects with T2DM.

**Methods:**

This cross-sectional study included 83 T2DM subjects without pre-existing cardiovascular diseases. CIMT was measured by color doppler ultrasonography, while serum Metrnl level was measured by Enzyme-linked Immunosorbent Assay (ELISA) assay. According to the CIMT status, the subjects were divided into two groups: T2DM without atherosclerosis (DM-AS, CIMT<1.0mm) and T2DM with atherosclerosis (DM+AS, CIMT ≥1.0mm or carotid plaque).

**Results:**

Serum Metrnl level was significantly increased in DM+AS group as compared to DM-AS group (0.77 ± 0.24 vs 0.51 ± 0.28 ng/ml, *P <*0.05). Binary logistic regression analysis showed that T2DM subjects in the fourth quartile of Metrnl levels demonstrated significantly higher risk of the presence of carotid atherosclerosis (*P <*0.05). The ROC curve used to identify the diagnostic accuracy of serum Metrnl level in the predication of T2DM subjects with carotid atherosclerosis revealed an area under the curve (AUC) of 0.742. The optimal cut-off value was 0.61 ng/ml with a sensitivity of 77.6% and a specificity of 58.8%.

**Conclusions:**

This cross-sectional study clearly demonstrated that elevated serum Metrnl level was significantly associated with higher risk of the presence of carotid atherosclerosis. Metrnl is a promising therapeutic target for T2DM and its macro-vascular diseases.

## 1 Introduction

Type 2 diabetes mellitus (T2DM) is strongly correlated with cardiovascular disease (CVD), as 70 - 80% of the mortality among T2DM patients is associated with microvascular and macrovascular complications (1). T2DM is a major cause of atherosclerosis and an independent risk factor for cardiovascular adverse events (2). Subclinical atherosclerosis, determined by carotid intimal medial thickness (CIMT) and the presence of carotid atherosclerotic plaque, is an independent predictor of cardiovascular risk (3). According to the ESC - EASD guidelines, CIMT measurement is a potential complementary method in cardiovascular risk assessment for diabetic patients (4).

Meteorin-like protein (Metrnl) is a newly discovered cytokine induced by exercise and cold exposure (5). It has been found to increase systemic energy expenditure, promote anti-inflammatory reactions, enhance insulin sensitivity in adipose tissue by regulating the peroxisome proliferator-activated receptor γ (PPARγ) signaling pathway (which improves glucose metabolism in diabetic models), and modulate immune - adipose interactions to drive anti-inflammatory gene expression, thus contributing to metabolic homeostasis (6). Several studies have reported elevated serum Metrnl levels in T2DM patients, linking its physiological role to the modulation of metabolic and inflammatory processes (7–9). However, the relationship between serum Metrnl levels and carotid atherosclerosis in T2DM remains unclear (10). Given the significance of early detection and prevention of cardiovascular complications in diabetes, our aim was to evaluate the association between serum Metrnl levels and carotid atherosclerosis, as determined by CIMT status in T2DM patients (11).

## 2 Materials and methods

### 2.1 Participants

A cross-sectional study was performed in T2DM subjects who were admitted to the Department of Endocrinology in the 900th Hospital of the Joint Logistics Team between October 2019 and August 2021. The inclusion criteria for the initial selection were: the patients met American Diabetes Association standards (version 2019) for T2DM (12), aged 35∼70 years, without pre-existing cardiovascular diseases. The criteria for exclusion were: type 1 diabetes, other specific types of diabetes, recent acute complication including diabetic ketoacidosis, hyperglycemic hyperosmolar state, cardiovascular diseases, liver or kidney failure, malignant tumors and current infectious conditions. Severe disability, severe malnutrition, or mental disorders were also excluded.

This study was conducted in accordance with the principles of the Declaration of Helsinki. The study was approved by the ethics committee of the Department of Endocrinology in the 900th Hospital of the Joint Logistics Team and informed consent was obtained from each participant before the examinations. A total 83 subjects with T2DM were evaluated. According to the CIMT status, the subjects were classified into two groups, the diabetes mellitus without atherosclerosis (DM-AS) whose CIMT was normal, while diabetes mellitus with atherosclerosis (DM+AS) including individuals with increased CIMT or plaque lesion. The analysis revealed a power of 82%, which, while not optimal, is still considered acceptable for detecting significant associations between serum Metrnl levels and carotid atherosclerosis in T2DM patients.

### 2.2 Carotid ultrasound evaluation of carotid atherosclerosis

Carotid artery ultrasound was a long-standing and reliable tool in the current armamentarium of diagnostic modalities used to assess vascular morbidity at an early stage (13). Carotid sonography was measured using the same color ultrasonography with a 7.0∼10 MHz transducer. The examinations were performed by the same trained and certified sonographers following the standard protocol, which was designed following guidelines from the American Society of Echocardiography consensus statement on subclinical vascular disease (14). All subjects were in supine position with the head slightly titled to the opposite side of the examination and titled back to fully expose the carotid arteries.

CIMT was defined as the vertical distance between the lumen-intima interface and media-adventitia interface, and it was measured at three points: the far wall of the mid and the distal common carotid artery, and 1.0 cm proximal to the carotid bulb. The mean value of the three measurements on each side was used to identify increased CIMT. A carotid plaque was defined as a focal wall thickening >50% of the surrounding IMT, or its CIMT ≥1.5 mm. The presence of carotid atherosclerosis was defined by an increase in CIMT above 0.9 mm and/or having carotid plaque (14).

### 2.3 Demographic characteristics

All participants submitted to physical measures using standard methods. Height and weight were obtained to calculate the body mass index (BMI) using a ratio of weight in kilogram (kg) to height in meters squared (m^2^). The participants’ blood pressure on the right arm with a mercury manometer was measured (OMRON HEM-7136) two times after sitting quietly for 5 minutes.

### 2.4 Laboratory measurements

Fasting venous blood samples for the laboratory measurements were collected using Vacutainer Ethylenediaminetetraacetic Acid (EDTA) tubes in the morning after 10-hour overnight fasting. Fasting blood glucose (FBG), creatinine (Cr), blood urea nitrogen (BUN), uric acid (UA) and lipid profiles including total cholesterol (TC), triglycerides (TG), low-density lipoprotein (LDL), and high-density lipoprotein (HDL) were measured using a fully automatic biochemical analyzer (Olympus AU2700). Glycated hemoglobin (HbA1c) levels were determined using the Variant TM device (Bio-Rad Variant II). Insulin was measured using the enzyme-linked immunosorbent assay (ELISA). Serum levels of METRNL and hypersensitive C reactive protein (hsCRP) were detected using an ELISA assay.

All the assays described above were performed according to the manufacturer’s instructions. Insulin resistance was calculated using the Homeostatic Model Assessment for Insulin Resistance (HOMA-IR) with the formula: FBG (mmol/L) * fasting insulin (mU/L)/22.5.

### 2.5 Statistical analysis

Statistical analyses were conducted using IBM SPSS Statistics (version 25) and GraphPad Prism (version 8). A *P*-value of < 0.05 (two-tailed) was considered statistically significant. Continuous variables were described using the mean and standard deviation (*SD*) for normally distributed data or the median and interquartile range (IQR, Q1–Q3) for skewed data. Categorical variables were expressed as frequencies and percentages. The Kolmogorov-Smirnov test was used to assess the normality of the data distribution. Normally distributed variables were compared between groups using independent samples *t*-tests, while skewed variables were analyzed using the Mann-Whitney U test (rank-sum test). Categorical variables were compared using Fisher’s exact test.

Spearman correlation analysis was used to evaluate the correlation between serum Metrnl level and other parameters. Serum Metrnl levels were transformed into ordinal grade variable by quartile segmentation method. Then binary logistic regression analysis was performed with CIMT status as a dependent variable to obtain the ORs and 95% CI for the risk of T2DM with AS by Metrnl quartile segmentations. Binary logistic regression model was done to obtain the odds ratios (ORs) and 95% confidence intervals (95% CI) for the risk of the presence of carotid atherosclerosis based on Metrnl quartile. Variables were included in the final model after verifying the absence of multicollinearity (Tolerance above 0.1 and VIF below 10).

Receiver operating characteristic (ROC) curve analysis was applied to identify the diagnostic accuracy of serum Metrnl level in the predication of T2DM subjects with carotid atherosclerosis. The cut-off values for serum Metrnl level that maximized the Youden index (sensitivity + specificity - 1) were defined as the optimal. The cut-off values that maximized the Youden index were defined as optimal. Youden index (sensitivity + specificity-1) is an integrative indicator of sensitivity and specificity.

## 3 Results

### 3.1 Comparisons between groups

The main demographic characteristics and laboratory measurements of the study groups were listed in **Table 1**. According to the level of CIMT status, the subjects were classified into two groups, T2DM without carotid atherosclerosis (DM-AS, CIMT < 1.0mm, *n*=34) and T2DM with carotid atherosclerosis (DM+AS, CIMT≥1.0mm or carotid plague, *n*=49). There were no significant differences in the proportion of gender, age, BMI, SBP, DBP, HbA_1_c, fasting plasma glucose (FPG), fasting serum insulin (FINS), HOMA-IR, lipid concentrations, including TC, TG, LDL-C, HDL-C, UA and hs-CPR between the two groups (*P*>0.05), indicating good comparability. However, there were significant differences in markers of kidney function between the two groups. Compared with the DM-AS group, the DM+AS group showed higher Cr and BUN level (*P <*0.05).

**Table 1:** Demographic characteristics and laboratory measurements of the two groups.

Variables	DM+AS (*n*=49)	DM-AS (*n*=34)	*t/Z/X^2^*	*P*
Gender (M/F)	37/12	19/12	2.686	0.101
Age (years, ˉx ± s)	59.04 ± 8.80	55.24 ± 10.62	1.779	0.079
BMI (kg/m^2^, ˉx ± s)	24.54 ± 3.77	25.20 ± 4.26	0.059	0.200
SBP (mmHg, ˉx ± s)	136.00 ± 18.97	130.53 ± 20.48	0.049	0.200
DBP (mmHg, ˉx ± s)	79.37 ± 10.54	80.76 ± 14.38	0.086	0.193
HbA1C [%, M(Q1,Q3)]	8.80 (7.55,10.45)	8.30(7.50,9.83)	0.909	0.380
FPG [mmol/L, *M*(*Q*1,*Q*3)]	7.35 (5.70,9.35)	7.61(5.87,10.57)	0.575	0.895
FINS [μIU/L, *M*(*Q*1,*Q*3)]	11.38 (7.60,17.36)	9.81(6.60,18.04)	0.645	0.799
HOMA-IR [*M*(*Q*1,*Q*3)]	3.82 (2.28,5.73)	3.18(1.52,6.06)	0.766	0.600
TC [mmol/L, *M*(*Q*1,*Q*3)]	4.26 (3.49,4.88)	4.68(3.84,5.59)	1.253	0.086
TG [mmol/L,*M* (*Q*1,*Q*3)]	1.64(1.04,2.16)	1.54(1.11,2.69)	0.667	0.765
LDL-C [mmol/L,*M*(*Q*1,*Q*3)]	2.63(2.09,3.35)	3.03(2.23,3.84)	1.345	0.054
HDL-C [mmol/L, *M*(*Q*1,*Q*3)]	1.01(0.74,1.21)	0.93(0.80,1.31)	0.702	0.708
Cr [mmol/L, *M*(*Q*1,*Q*3)]	81.00(63.8,125.50)	63.60(56.78,79.30)	1.538	0.018^*^
BUN [μmol/L,*M*(*Q*1,*Q*3)]	6.30(5.10,7.85)	5.00(3.88,6.45)	1.396	0.041^*^
UA [μmol/L,*M*(*Q*1,*Q*3)]	345.30(263.05,408.65)	303.65(214.93,404.90)	1.111	0.170
hsCPR [mg/L, *M*(*Q*1,*Q*3)]	2.30(0.70,8.35)	1.60(0.45,4.48)	0.847	0.470

BMI, Body mass index; SBP, systolic blood pressure; DBP, diastolic blood pressure; HbA_1c_, hemoglobin A1c; FPG, fasting plasma glucose; FINS, fasting insulin; HOMR-IR, Homeostasis model assessment insulin resistance index; TC, total cholesterol; TG, triglyceride; LDL-C, Low-density lipoprotein-cholesterol; HDL-C, high-density lipoprotein-cholesterol; BUN, blood urea nitrogen; Cr, creatinine; UA, uric acid; hsCRP, hypersensitive C reactive protein.* *P <*0.05 vs DM-AS group.

### 3.2 Comparison of serum Metrnl levels between the DM-AS group and the DM+AS group

**Figure 1:**
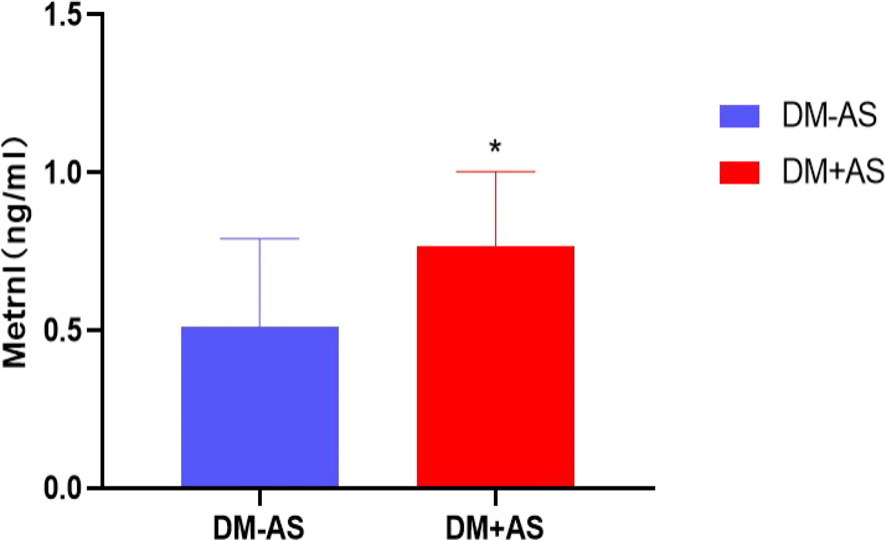
Comparison of serum Metrnl levels between the DM-AS group and the DM +AS group. ^*^ *P* < 0.05 vs the DM +AS group.

The study showed that serum Metrnl levels were significantly increased in the subjects with carotid atherosclerosis as compared to those without carotid atherosclerosis (0.77 ± 0.24 vs 0.51 ± 0.28 ng/ml) with *P <*0.05 (**Figure 1**).

### 3.3 Spearman correlation analysis of serum Metrnl level with other variables

Spearman correlation analysis was used to assess the relationship between serum Metrnl level and other variables. Results indicated that serum Metrnl level was correlated positively with CIMT status (r=0.413*, P*<0.05) and Cr (r=0.367, P<0.05) (**Table 2**). Whereas, Serum Metrnl level was not significantly correlated with gender, age, BMI, SBP, DBP, HbA_1C_, FPG, FINS, HOMA-IR, TC, TG, LDL-C, HDL-C), BUN and UA and hsCPR (All *P >*0.05, **Table 2**).

**Table 2:** Spearman correlation analysis of serum Metrnl level and other variables.

Variables	*r*	*P*
Gender	-0.075	0.502
Age	0.111	0.319
BMI	-0.066	0.556
SBP	0.096	0.390
DBP	0.130	0.241
HbA_1C_	-0.039	0.729
FPG	-0.151	0.174
FINS	-0.034	0.762
HOMA-IR	-0.058	0.605
TC	-0.094	0.398
TG	0.039	0.727
LDL-C	-0.162	0.143
HDL-C	-0.117	0.293
Cr	0.367	0.001*
BUN	0.180	0.103
UA	0.201	0.068
hsCPR	0.109	0.325
CMIT status	0.413	0.000*

Statistically significant at * *P <*0.05.

### 3.4 Multiple regression analysis for CIMT and serum Metrnl levels

Serum Metrnl levels were transformed into ordinal grade variable by quartile segmentation method. Then binary logistic regression analysis was performed with CIMT status as a dependent variable to obtain the ORs and 95%CI for the risk of T2DM with AS by Metrnl quartile segmentations. The study showed that T2DM subjects in the fourth quartile of Metrnl levels demonstrated significantly higher risk of the presence of carotid atherosclerosis even after adjusting for demographic characteristics and factors which were associated with serum Metrnl level (All *P <*0.05, **Table 3**).

**Table 3:** The ORs and 95%CI of for the risk of the presence of carotid atherosclerosis in T2DM based on Metrnl quartile.

Model	Metrnl quartile segmentations				
	Q1	Q2	Q3	*Q4*	*P*
unadjucted	Ref.	2.344(0.661, 8.305)	3.281(0.968, 11.125)	6.625(2.377, 9.503)	0.002^*^
Model 1	Ref.	2.458(0.63, 9.589)	3.770(0.936, 15.175)	5.494(2.290, 8.181)	0.004^*^
Model 2	Ref.	2.453(0.629, 9.563)	3.881(0.949, 15.865)	13.540(2.262, 8.053)	0.004 ^*^
Model 3	Ref.	2.420(0.786, 9.583)	3.890(0.900, 15.809)	6.900(2.569, 8.053)	0.004 ^*^

Model 1: Adjusted for age, gender, BMI, smoking, alcohol.

Model 2: Adjusted for variables in Model 1 and SBP, DBP, Cr, LDL.

Model 3: Adjusted for variables in Model 2 and hypertension.

^*^ *P <*0.05 vs Q1.

**Table 4** presented the estimated associations between c-IMT status and several potential confounding factors, expressed as *OR* with corresponding 95% *CI*. Male individuals exhibited a higher likelihood of an elevated c-IMT compared to females (*OR* = 1.386, 95% *CI*: 1.259–1.498, *P* = 0.001). The odds ratio for age was 1.187, with a *P* value of 0.287, suggesting no potential association. Current smokers had a significantly increased risk of elevated c-IMT compared to non-smokers (*OR* = 1.189, 95% *CI*: 1.079–1.246, *P* = 0.011). Individuals who consumed alcohol showed a significantly higher risk of elevated c-IMT compared to non-drinkers (*OR* = 1.289, 95% *CI*: 1.127–1.479, *P* = 0.021). The association between BMI and c-IMT status was not significant (*OR* = 1.369, 95% *CI*: 0.985–1.659, *P* = 0.795). Higher levels of Metrnl were associated with an increased likelihood of elevated c-IMT (*OR* = 1.278, 95% *CI*: 1.067–1.487, *P* = 0.037). These findings suggest that male sex, smoking, alcohol consumption, and higher Metrnl levels are significant risk factors for increased c-IMT, while age and BMI did not show statistically significant associations.

**Table 4:** Estimated association of c-IMT status and potential confounding factors.

	*OR*	*OR* 95% *CI*	*p* value
Sex			
Female	Ref.	—	—
Male	1.386	1.259, 1.498	0.001 ^*^
Age	1.187	0.987, 1.218	0.287
Smoking			
No	Ref.	—	—
Yes	1.189	1.079, 1.246	0.011 ^*^
Alcohol			
No	Ref.	—	—
Yes	1.289	1.127, 1.479	0.021 ^*^
BMI	1.369	0.985, 1.659	0.795
Metrnl	1.278	1.067, 1.487	0.037 ^*^

^*^ *P* < 0.05.

### 3.5 The diagnostic accuracy of serum Metrnl level in the predication of T2DM subjects with carotid atherosclerosis

**Figure 2:**
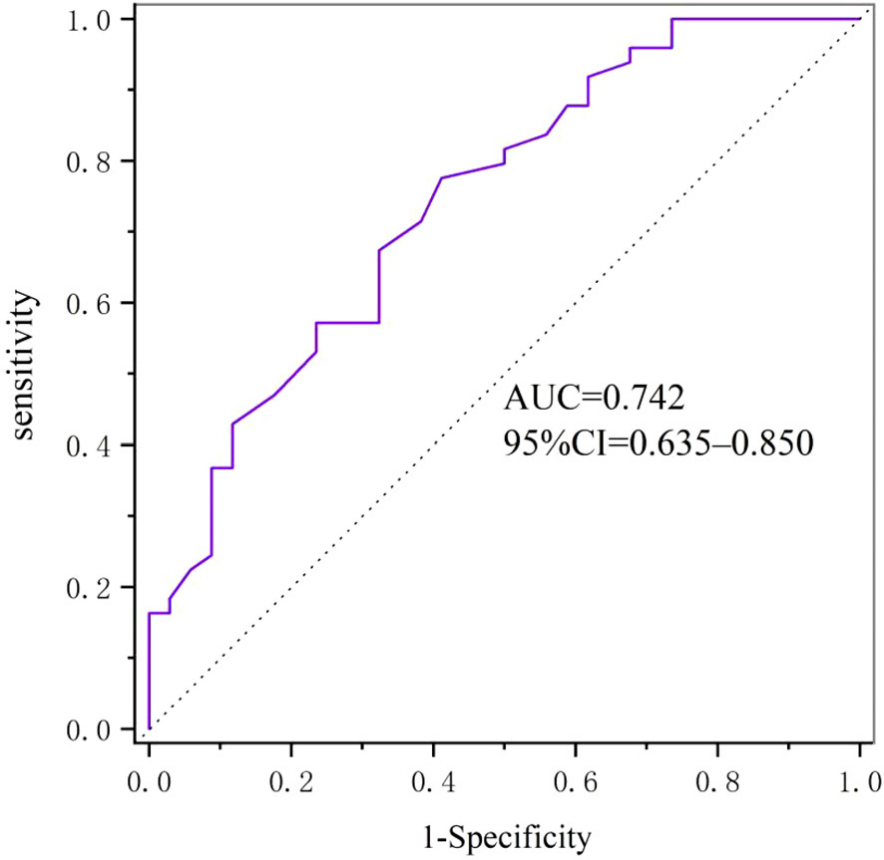
Diagnostic accuracy of serum Metrnl level in the predication of T2DM subjects with carotid atherosclerosis evaluated by ROC curve.

ROC curve analysis was used to identify the diagnostic accuracy of serum Metrnl level in the predication of T2DM subjects with carotid atherosclerosis. The ROC curve revealed an area under the curve (AUC) of 0.742 for serum Metrnl level. The optimal cut-off value of serum Metrnl level that predicted T2DM with AS was 0.61 ng/ml, and the sensitivity and specificity were 77.6% and 58.8%, respectively (**Figure 2**).

## 4 Discussions

The global diabetes prevalence in 20∼79 years old in 2021 was estimated to be 10.5% (536.6 million people), rising to 12.2% (783.2 million) in 2045 (15). People living with diabetes are at risk of developing several life-threatening complications, including micro- and macro-vascular complications, leading to an increased need for medical care, reduced quality of life and premature death (16–18). Global diabetes-related health expenditures were estimated at 966 billion USD in 2021, and are projected to reach 1,054 billion USD by 2045 (19). T2DM is the most common type of diabetes, accounting for more than 90% of all diabetes worldwide. CAPTURE study found that the prevalence of cardiovascular disease (CVD) in adults with T2DM is approximately 1 in 3 17. Both CAPTURE study and Chinese Da Qing study showed that CVD was the predominant cause of death in those with T2DM (19, 20). It is estimated that over 6.7 million people aged 20∼79 will die from diabetes-related causes in 2021. In April 2021, the World Health Organization launched its Global Diabetes Compact, marking an increased focus on diabetes and their complications (21).

Two distinct measures of carotid atherosclerosis are carotid plaque burden, quantified by CIMT, and the presence of plaques (22, 23), and they are highly correlated with cardiovascular disease. It was reported that carotid atherosclerosis was an independent risk factor for 10-year coronary heart disease risk (24). CIMT measurement was useful for risk assessment of cardiovascular diseases and it was a strong predictor of future cardiovascular and cerebral events (25). CIMT has been found to predict future risk of myocardial infarction and stroke. The increase in CIMT has been reported as an independent marker for cardiovascular risk and event (24, 25). In this cross-sectional study, according to the level of CIMT status, the subjects were classified into two groups, T2DM without carotid atherosclerosis (DM-AS, CIMT<1.0mm, n=34) and T2DM with carotid atherosclerosis (DM+AS, CIMT≥1.0mm or carotid plague, n=49). There was no statistically significant difference in the main demographic characteristics and laboratory measurements between the study groups, indicating good comparability.

Several studies have reported that Metrnl induced by exercise and cold exposure played an important role in antagonizing insulin resistance (5, 6, 9, 26). Metrnl in adipose tissue regulated insulin sensitivity via the PPARƳ pathway, and PPARƳ expression in adipose tissue was dramatically up-regulated by Metrnl *in vitro* and *in vivo*. Furthermore, both inhibition and knockdown of PPARƳ prevent the Metrnl mediated improvement of insulin resistance (6). Metrnl can stimulate the anti-inflammatory gene expression and improve the glucose metabolism of obese diabetic mice. Li et al. found that knockout of Metrnl gene in adipose tissue may worsen insulin resistance, and over expression of Metrnl gene in adipose tissue can prevent insulin resistance in mice (9). However, the association between the serum Metrnl level and carotid atherosclerosis subjects with T2DM is still unclear. This study demonstrated firstly that serum Metrnl concentration was distinctly elevated in the subjects with carotid atherosclerosis as compared to those without carotid atherosclerosis (0.77 ± 0.24 vs 0.51 ± 0.28 ng/ml, P <0.05). Moreover, binary logistic regression analysis performed with CIMT status as a dependent variable showed that T2DM subjects in the fourth quartile of Metrnl levels demonstrated significantly higher risk of the presence of carotid atherosclerosis even after adjusting for demographic characteristics and factors which were associated with serum Metrnl level. We speculate that higher serum Metrnl level had an additive effect on the presence of carotid atherosclerosis in subjects with T2DM. Serum Metrnl level may be a useful indicator of carotid atherosclerosis in subjects with T2DM. The ROC curve used to identify the diagnostic accuracy of serum Metrnl level in the predication of T2DM subjects with carotid atherosclerosis revealed good diagnostic accuracy with an area under the curve (AUC) of 0.742. The optimal cut-off value was 0.61 ng/ml with a sensitivity of 77.6% and a specificity of 58.8%. It demonstrated a positive correlation between the serum Metrnl and the Cr level in subjects with T2DM. The results of Lee et al., as well as the study between serum Metrnl level and visceral fat obesity (VFO) in T2DM were consistent with this study. They indicated that Metrnl is a promising therapeutic target for T2DM and its micro- and macro-vascular diseases.

This study also had some limitations. First, because of the inherent limitations of the cross-sectional study design, we could not clarify whether a causal relationship exists between serum Metrnl level and carotid atherosclerosis in T2DM patients. Second, we only investigated this relationship in subjects with T2DM, thus the findings require validation in other types of diabetes and healthy subjects. And in this study, we could not fully exclude other potential confounding factors, especially exercise and exposure to cold temperature. Third, we are confident that including a control group of participants with normal glucose tolerance would be highly beneficial, while unfortunately, it was difficult for us to find such a control. future studies will include a control group to further validate these findings in subjects with normal glucose tolerance. Forth, it likely stems from our relatively small sample size, which may have failed to capture the full range and variability of the population. This could potentially lead to less precise estimates and limit the generalizability of our findings. We are aware that this may affect the strength of our conclusions and the interpretation of the data. To overcome this limitation, we plan to conduct a follow-up study with a significantly larger sample size. By increasing the number of participants, we expect to obtain more accurate and stable results, which will help to better understand the underlying relationships and phenomena under investigation. This will also allow us to provide more robust evidence and more conclusive insights in future research.

## 5 Conclusions

This cross-sectional study clearly demonstrated that elevated serum Metrnl level was significantly associated with higher risk of the presence of carotid atherosclerosis, independent of the conventional cardiometabolic risk factors. Serum Metrnl level may be a useful indicator of carotid atherosclerosis in subjects with T2DM. Metrnl is a promising therapeutic target for T2DM and its macro-vascular diseases.

## Data Availability

The raw data supporting the conclusions of this article will be made available by the authors, without undue reservation.
